# Seroprevalence of Hepatitis A Virus Antibodies among the Patients with Chronic Hepatitis B in Turkey

**DOI:** 10.5005/jp-journals-10018-1143

**Published:** 2016-07-09

**Authors:** Necla Tulek, Metin Ozsoy, Cigdem Moroglu, Meliha Cagla Sonmezer, Fatih Temocin, Gunay Tuncer Ertem, Fatma Sebnem Erdinc

**Affiliations:** 1 Department of Infectious Diseases and Clinical Microbiology, Ankara Training and Research Hospital, Ankara, Turkey

**Keywords:** Chronic hepatitis B, Hepatitis A infection, Seroprevalence, Vaccination.

## Abstract

**Background:**

Hepatitis A virus (HAV) can cause significant pathology in patients with chronic hepatitis B virus (HBV), however, HAV can be prevented by vaccination. The aim of this study was to determine the implication of vaccination against HAV vaccine in patients with chronic hepatitis B.

**Materials and methods:**

The seroprevalence of anti-HAV IgG antibodies was investigated in the patients with chronic hepatitis B. Anti-HAV IgG antibodies were detected by commercially available ELISA kit.

**Results:**

A total of 673 patients (354 males, 319 females with age range of 17-78 years) with chronic hepatitis B were included the study. Hepatitis A virus seropositivity rate was 34% in the patients younger than 20 years, 79% in the age group of 20 to 29 years, and 100% after 35 years of age.

**Discussion:**

Hepatitis A virus vaccination may be recommended for young adult patients with chronic hepatitis B in Turkey.

**How to cite this article:**

Tulek N, Ozsoy M, Moroglu C, Sonmezer MC, Temocin F, Ertem GT, Erdinc FS. Seroprevalence of Hepatitis A Virus Antibodies among the Patients with Chronic Hepatitis B in Turkey. Euroasian J Hepato-Gastroenterol 2015;5(2):95-97.

## INTRODUCTION

Hepatitis A is an acute contagious diseases caused by a single stranded RNA virus belong to Picarnoviridae family. While most of infections are asymptomatic in childhood, it is symptomatic in three quarters of adults and causes to icteric acute hepatitis. It is generally benign diseases, prognosis is generally good and it leaves long-term immunity. On the other hand, it may progress to fulminant hepatic failure and may cause to death in rare cases and liver transplantation may be required. Mortality rate increases with age and mortality is also higher among the patients with chronic liver diseases.^1^ Although, hepatitis A vaccination is recommended as a part of management of chronic hepatitis B, more insights are required in this context.

Turkey is intermediately endemic for hepatitis B virus (HBV) infection and overall prevalence rate is approximately 4.6%.^2^ The seroprevalence of hepatitis A virus (HAV) has been changing according to age, region and socioeconomic conditions. About half of adolescents and more than 90% of adults are immune to HAV.^3^ Both of hepatitis A and B are vaccine preventable diseases. Hepatitis B vaccination has been introduced to newborns since 1998 in Turkey. Recently (in 2012) hepatitis A vaccine was also implemented in routine childhood vaccination by Turkish Ministry of Health.

In this study, hepatitis A seroprevalence was investigated in patients with chronic hepatitis B to determine the susceptibility and requirement of hepatitis A vaccine in different age groups.

## MATERIALS AND METHODS

This study was conducted in patients with chronic HBV infection at Department of Infectious Diseases and Clinical Microbiology of Ankara Training and Research Hospital between 2009 and 2013 years. Patients who were positive for hepatitis B surface antigen (HBsAg) for more than 6 months were included in this study. The medical records of the patients, and test results were reviewed retrospectively. The patients who were vaccinated with hepatitis A vaccine were excluded from the study. Initial routine HAV IgG test results of the patients when they admitted to our outpatient clinic were taken in consideration. Anti-HAV Ig antibodies were detected in sera of patients by a commercially available Microparticle Enzyme Immunoassay (MEIA; AxSYM-HAVAB 2.0, ABBOTT). The HAV IgG positivity rates were evaluated and compared between the age groups.

The statistical analysis was performed using a SPSS version 15.0 program. Student’s t-test and Chi-squared test were used to compare the differences in the seroprevalence rates and a significant difference was considered when p < 0.05.

## RESULTS

**Graph 1:**
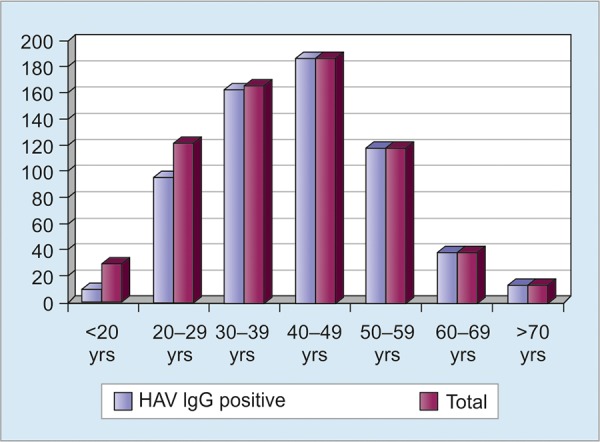
Anti-HAV IgG seroprevalence rate among the patients with chronic hepatitis B

A total of 673 patients (354 males, 319 females with an age range of 17-78 years) were included the study. Most of the patients with chronic hepatitis B (53%) were in 30 to 49 years age group. Overall anti-HAV IgG seropositivity rate was 90.5% in the study population. The distribution of patients according to age groups and anti-HAV IgG positivity rates were shown in Graph 1. Low anti-HAV IgG seropositivity rate (34%) was detected in the age group younger than 20 years old. The seroprevalence rate was increasing with age and it reached 100% at the age of > 40 years. All of the patients older than 35 years had been encountered HAV and expressing anti-HAV IgG. There was no significant difference in anti-HAV IgG positivity according to gender.

## DISCUSSION

Vaccination, infrastructure, sanitation, and socio-economical level are major parameters effecting HAV seroprevalence in a community. Turkey is a moderately endemic country for HAV infection with over all prevalence rates above 90%. In recent years, due to improvement in infrastructural and sanitary conditions, epidemiology of HAV is changing.^4,5^ The pattern of acute HAV infection is shifting from childhood infection to adolescent and young adult infection. Hepatitis A virus seroprevalence may change within countries even in regions. Our hospital is located in the Central Anatolia region. In this study, seroprevalence rate of HAV was found as 34% in the age group younger than 20 years old. The seroprevalence rates were reported between 87 and 43% at the same region in the same age groups previously.^6-8^ Seropositivity of HAV for the age group of 20 to 29 years was about 67 to 91% in these studies; it was 79% in our study. Although, 100% of patients over 40 years have been exposed to virus, this study shows that HAV seropositivity is declining in young population.

Hepatitis A is a self-limiting disease generally, but it may cause more severe disease and worse outcome if it is superimposed on chronic hepatitis B.^9-11^ Hepatitis A vaccine is available for more than 20 years and effective for pre-exposure prophylaxis of hepatitis A in susceptible individuals.^12^ Routine vaccination of children is recommended over 1 year of age.^13^ Vaccine is also recommended to high risk groups like people with chronic liver disease, receiving clotting factor concentrates, working with virus, traveling to highly endemic countries, and during the outbreaks.^14^ In Turkey routine vaccination of children with inactive hepatitis A vaccine was initiated in 2012. In the future, hepatitis A infection will be under control. But until that, routine screening of patients particularly less than 40 years old with chronic HBV infection should be checked for HAV prevalence and should be vaccinated accordingly.
